# Multi-omics analysis reveals the alleviating effect of oxidation remediation on tobacco quinclorac stress

**DOI:** 10.3389/fmicb.2025.1625585

**Published:** 2025-09-15

**Authors:** Binghui Zhang, Ting Yang, Chenliang Cheng, Tong Li, Ni Zhang, Fei Wang, Wencan Chen, Zhiping Zhong, Zhaoxiang Liu, Gang Gu, Xiangmin Lin, Xiaofang Xie

**Affiliations:** ^1^College of JunCao Science and Ecology, College of Life Sciences, Fujian Agriculture and Forestry University, Fuzhou, China; ^2^Institute of Tobacco Science, Fujian Provincial Tobacco Company, Fuzhou, China; ^3^Jianning Branch of Sanming Tobacco Company, Sanming, China; ^4^Changting Branch of Longyan Tobacco Company, Longyan, China; ^5^Fujian Key Laboratory of Crop Breeding by Design, Fujian Agriculture and Forestry University, Fuzhou, China

**Keywords:** muti-omics, K_2_S_2_O_8_, C_10_H_5_C_2_NO_2_, oxidation repair, tobacco

## Abstract

The extensive use of the herbicide quinclorac has led to significant residues in agricultural soil, posing adverse effects on crop safety and high-quality production. In this study, using the tobacco variety CB-1 as material, we found that oxidizing agent K_2_S_2_O_8_ can significantly reduce quinclorac-induced phytotoxicity symptoms in tobacco. Furthermore, we integrated biochemical methods, metagenomics, metabolomics, and transcriptomics to investigate the effects of K_2_S_2_O_8_ on both quinclorac-contaminated soil and tobacco plants. Soil physicochemical properties analysis showed that the incorporation of K_2_S_2_O_8_-based remediation significantly mitigated the negative effects of quinclorac and largely restored the soil properties affected by quinclorac stress. Metagenomic analysis found that quinclorac significantly reduced soil species diversity, while K_2_S_2_O_8_-based remediation soil exhibited higher richness of microbial communities, with increased abundance of *Sphingomonas* and *Bradyrhizobium*, and decreased abundance of *Alphaproteobacteria*. Differential gene expression analysis showed significant up-regulation and down-regulation of genes under C_10_H_5_Cl_2_NO_2_ stress, which was partially mitigated by K_2_S_2_O_8_ treatment. Gene Ontology (GO) enrichment analysis indicated that these genes were mainly involved in cellular processes, metabolic pathways, and biological regulation. Metabolomic analysis further confirmed significant changes in metabolite profiles, with K_2_S_2_O_8_ treatment restoring many metabolites to near control levels. Integrated metabolomic-transcriptomic analysis revealed enrichment of differentially expressed genes (DEGs) and metabolites in six key pathways: (1) lysine degradation, (2) stilbenoid diarylheptanoid and gingerol biosynthesis, (3) arginine and proline metabolism, (4) phenylalanine biosynthesis, (5) tyrosine metabolism, and (6) flavonoid biosynthesis. Additionally, the levels of 4-hydroxyphenylacetylglutamic and 5-aminovaleric acid were down-regulated, along with the expression of genes associated with these metabolites, when quinclorac residual soil was treated by K₂SO_8_. The results of this study provide a theoretical basis for the remediation of pesticide residue soil in rice tobacco rotation areas, offering valuable insights for sustainable agricultural practices.

## 1 Introduction

Herbicides play an important role in reducing weed damage and promoting global food production security in current agricultural practices (Li et al., 2024). Among them, quinclorac with the molecular formula C_10_H_5_Cl_2_NO_2_ is a growth hormone-like herbicide known for its strong selectivity and long persistence, widely used to control monocotyledonous weeds in rice fields (Kieling and Pfenning, 1990). However, due to its relatively stable structure, quinclorac is difficult to degrade and prone to residue in acidic soils of southern regions, which may cause phytotoxicity on subsequent crops (Zang et al., 2020), especially on Solanaceous crops such as tobacco, potato, tomato, and eggplant (Qiuzan et al., 2018). In the rotation areas of tobacco-rice cultivation in southern China, quinclorac leads to leaf deformities (such as curling or narrowing) in tobacco plants, affecting both yield and quality of tobacco leaves and causing significant economic losses for farmers (Huang et al., 2021). Therefore, it is urgent to find solutions for alleviating the toxicity caused by quinclorac.

Soil remediation is considered the primary method for reducing the phytotoxicity of quinclorac on tobacco. The remediation process for land pollution can be categorized into two types: *in situ* and ex situ (Marcon et al., 2021). *In situ* remediation directly treats pollution sources without extra costs, making it the optimal choice. It encompasses three key strategies: bioremediation, physical remediation, and chemical oxidation remediation (Bains et al., 2019; He et al., 2015; Sun et al., 2017; Yu et al., 2019). Physical remediation has high costs and labor intensity. Additionally, when adsorbents reach the saturation point over time it leads to pesticide residues accumulating and losing their effectiveness (Dermont et al., 2008). In contrast, chemical oxidation remediation shows great potential in dealing with emerging pollutants. The oxidants used include ozone, Fenton reagent, potassium permanganate (KMnO4), and persulfate (Zeng et al., 2016). Among them, persulfate exhibits a higher redox potential resulting in longer lifespan during reactions with organic pollutants while facilitating better contact with pollutants (Yen et al., 2011). It has been successfully applied in degrading various pollutants such as PAHs (polycyclic aromatic hydrocarbons), PBDEs (polybrominated diphenyl ethers), PNP (p-nitrophenol), and atrazine (Chen et al., 2016, 2018; Peng et al., 2017; Song et al., 2019). To date, there have been no reports on the use of the oxidizing agent K_2_S_2_O_8_ for the remediation of soil contaminated with quinclorac.

Non-biological stressors such as pesticides can simultaneously induce changes in crop rhizosphere microbiota (Daniel and Bernot, 2014), metabolites (Urano et al., 2010), and related genes. The utilization of multi-omics analysis techniques combining rhizosphere microbiome, metabolome, and transcriptome is an effective method for exploring the mechanisms underlying plant stress alleviation. Wu et al. (2021) successfully applied this approach in studying cucumber response to hydroxybenzoic acid stress. However, there have been no reports on the application of multi-omics analysis techniques to investigate the mechanism by which sulfate mitigates quinclorac-induced damage in tobacco leaves. In this study, we found that oxidizing agent K_2_S_2_O_8_ can significantly reduce phytotoxicity symptoms of tobacco induced by quinclorac. To explore the underlying mechanisms, we integrated biochemical methods, rhizosphere microbiota, metabolome, and transcriptome to investigate the effects of persulfate on both quinclorac-contaminated soil and the tobacco plants. This included examining changes in soil characteristics and soil microbial community, and the expression and metabolism of tobacco plant. The results of this study will provide a foundation for the remediation of herbicide residues in soil within rice-tobacco rotation areas.

## 2 Materials and methods

### 2.1 Plant materials and treatments

The main tobacco variety CB-1 in the tobacco-growing area of Fujian province was used as experimental material. The pot experiment was conducted from April to June 2024 at the Fujian Key Laboratory of Crop Breeding by Design, situated within the greenhouse of Fujian Agriculture and Forestry University. The greenhouse environment was maintained at a 16-h day temperature of 22 °C and an 8-h night temperature of 18 °C. The experimental design comprised a control group (denoted as CK) without the addition of either C_10_H_5_Cl_2_NO_2_ or K_2_S_2_O_8_ to the soil. The treatment groups included soil added with 0.04 mg/kg C_10_H_5_Cl_2_NO_2_ (denoted as C), and soil amended with both 0.04 mg/kg C_10_H_5_Cl_2_NO_2_ and 100 mg/kg K_2_S_2_O_8_ (denoted as CY). Each treatment involved five plants, with three biological replicates for a total of 45 pots.

### 2.2 Sample collection

The samples were collected at 45 days post-treatment. This included soil samples for soil characteristics, rhizospheric soil for microbial community analysis, and tobacco leaves for gene expression and metabolic profiling of the tobacco plants. For the microbial community analysis, soil samples within a range of 1–4 mm around the roots of the three treatment groups were collected, with approximately 100 g per treatment group. Additionally, 100 g of soil was collected from five pots per treatment group for soil characteristics analysis. For gene expression and metabolic profiling of tobacco, leaves in the 2nd–3rd positions from the top (counting from the uppermost leaf) were selected for this study. A total of ten leaves, sourced from five different plants, were collected as a sample. All samples were collected with three biological replicates. Following collection, the soil samples were stored at −20 °C, while the leaf samples were stored at −80 °C until analysis.

### 2.3 Investigation of soil physical and chemical properties

The collected soil samples were initially purified to remove impurities, and then passed through a 2 mm mesh for homogenization. The air-dried soil samples were subsequently analyzed for their properties and nutrient content. The organic matter was analyzed according to the NY/T1121.6-2006 method; pH was determined using the NY/T1377-2007 method; total nitrogen level was measured following the NY/T53-1987 method; total phosphorus content was assessed based on the NY/T88-1988 method; total potassium concentration was determined according to the NY/T87-1988 method; available nitrogen were evaluated using the method described in LY/T 1228-2015; available phosphorus levels were evaluated using the NY/T1121.7-2006 method; and available potassium concentration was measured following the NY/T889-2004 method. Soil particle size measurements were carried out according to the NY/T1121.3-2006 method.

### 2.4 Metagenomic analysis reveals changes in soil microbial communities

The high-throughput metagenomic sequencing technology was used to investigate changes in soil microbial communities under three treatments. Initially, microbial DNA was extracted and purified from soil samples using a bacteria and fungi genomic extraction kit (Omega D3350-02; Solarbio D2300-100T). The resulting DNA fragments were generated through ultrasound treatment, followed by purification, end-repair, 3′-end adenylation, and ligation with sequencing adapters. Subsequently, agarose gel electrophoresis was employed to select appropriately-sized fragments for PCR amplification library construction. Metagenome sequencing was performed on the Illumina Hiseq2500 platform following standard protocols. After data processing and statistical analysis, including low-quality data filtering, output data generation, and quality control statistics, the metagenome assembly was carried out using MEGAHIT software while QUAST software (Tang and Borodovsky, 2014) evaluated the assembly results by removing contig sequences shorter than 300 bp. Additionally, MetaGeneMark software was used for coding region identification and removal of redundant data. Finally, prediction analysis of tobacco rhizosphere microbial community structure and alpha diversity under different treatments was conducted on BMK Cloud.^1^

### 2.5 Transcriptomic analysis

RNA-Seq was used for the treatments and their control to investigate the potential mechanism underlying K_2_S_2_O_8_-mediated C_10_H_5_Cl_2_NO_2_ stress mitigation. Total RNA of samples (C, Y, CK) was extracted using the TRIzol reagent (Invitrogen, USA). RNA sequencing (RNA-Seq) and data processing were performed with the Illumina HiSeq platform at Biomarker Technologies Co., LTD. (Beijing, China) according to Cho et al. (2016). The RNA-Seq data have been submitted in the NCBI Sequence Read Archive (SRA) under the accession number PRJNA1221589.

After excluding reads containing adapter, poly-N, and low-quality sequences, the remaining clean reads were aligned to the reference genome in Sol Genomics Network database.^2^ Subsequently, these aligned reads were assembled and quantitatively analyzed using StringTie software to determine the fragments per kilobase of exon per million fragments mapped (FPKM) values. DEGs were identified using a false discovery rate (FDR) ≤ 0.01 and |Fold change| ≥ 1.5 while calculating FDR and Fold change (FC) for all genes. Additionally, GO and Kyoto Encyclopedia of Genes and Genomes (KEGG) enrichment analyses were performed for the three comparison groups: CK vs. C, CK vs. CY, and C vs. CY.

### 2.6 Widely targeted metabolomics analysis

The metabolites of leaf samples at 45 days post-treatment (C, CY, and CK) were analyzed using widely targeted metabolomics methods. Freeze-dried leaves were homogenized using a mixer mill (MM 400, Retsch, Germany), and the leaf powder was pooled from each biological replicate sample, 100 mg of this powder was extracted overnight at 4 °C with 0.6 mL of 70% aqueous methanol. The extracts were subjected to analysis using ultra-performance liquid chromatography with electrospray ionization coupled to tandem mass spectrometry (UPLC–ESI–MS/MS) at Biomarker Technologies Co., LTD. (Beijing, China).

Metabolic data from each sample were analyzed using hierarchical cluster analysis (HCA), principal component analysis (PCA), and K-means clustering. HCA and PCA analyses were performed using Software R and GraphPad Prism v9.01 (GraphPad Software Inc., La Jolla, CA, USA), respectively. DEMs among samples from different groups were identified based on the following criteria: VIP ≥ 1, |Fold change| ≥ 1, and *p* value <0.01. The Venn diagram illustrates the quantitative relationship among different comparison groups. The Kyoto Encyclopedia of Genes and Genomes (KEGG) compound database^3^ was utilized for annotating the different metabolites which were then mapped onto the KEGG pathway database.^4^ Pathways containing significantly regulated metabolites underwent further analysis through metabolite sets enrichment analysis (MSEA). Significance assessment was conducted by calculating *p*-values obtained from hypergeometric tests.

### 2.7 Integrated multi-omics analysis

The Spearman test method (Heinen and Valdesogo, 2020) was employed to conduct correlation analysis among metabolomics, transcriptomics, and microbiota. Results meeting the criteria of a *p*-value <0.05 and a Spearman correlation coefficient |*r*| > 0.8 were chosen for constructing a correlation network.

### 2.8 Quantitative real-time PCR (qRT-PCR) analysis

Total RNA was isolated from plantlets using TRIzol reagent (Invitrogen) according to the manufacturer’s protocol. The extracted RNA was then reverse-transcribed into complementary DNA (cDNA), which was used for quantitative real-time PCR (qRT-PCR) analysis with SYBR Premix ExTaq (Takara). The expression of the Actin gene was employed as an internal control. The experiment was conducted with three biological replicates, each comprising three individual plants, and each sample was analyzed in triplicate. The relative gene expression levels were determined using the 2^−ΔΔCt^ method (Livak and Schmittgen, 2001), and the primer sequences used for qRT-PCR are provided in Supplementary Table 1.

## 3 Results

### 3.1 Oxidizing agent K_2_S_2_O_8_ reduces phytotoxicity symptoms in tobacco induced by C_10_H_5_Cl_2_NO_2_ herbicides

**Figure 1:**
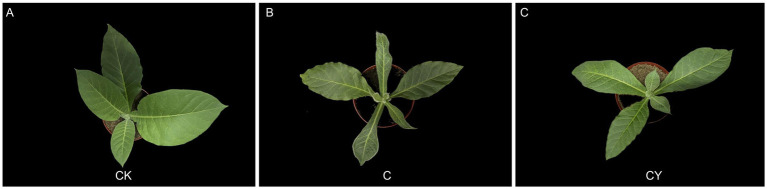
The morphology of tobacco leaves under three treatment. **(A)** Control soil without the addition of C_10_H_5_Cl_2_NO_2_ or K_2_S_2_O_8_. **(B)** The soil was treated with 0.04 mg/kg C_10_H_5_Cl_2_NO_2_. **(C)** The soil was treated with 0.04 mg/kg C_10_H_5_Cl_2_NO_2_ and 100 mg/kg K_2_S_2_O_8_.

In comparison to the control (CK) (Figure 1A), tobacco seedlings exposed to C_10_H_5_Cl_2_NO_2_ herbicides exhibited leaf curling/narrowing and stunted growth (Figure 1B). Notably, K_2_S_2_O_8_ treatment significantly alleviated these symptoms (Figure 1C), suggesting its potential role in mitigating herbicide-induced damage.

### 3.2 Impacts of C_10_H_5_Cl_2_NO_2_ and K_2_S_2_O_8_ on soil physicochemical properties

**Figure 2:**
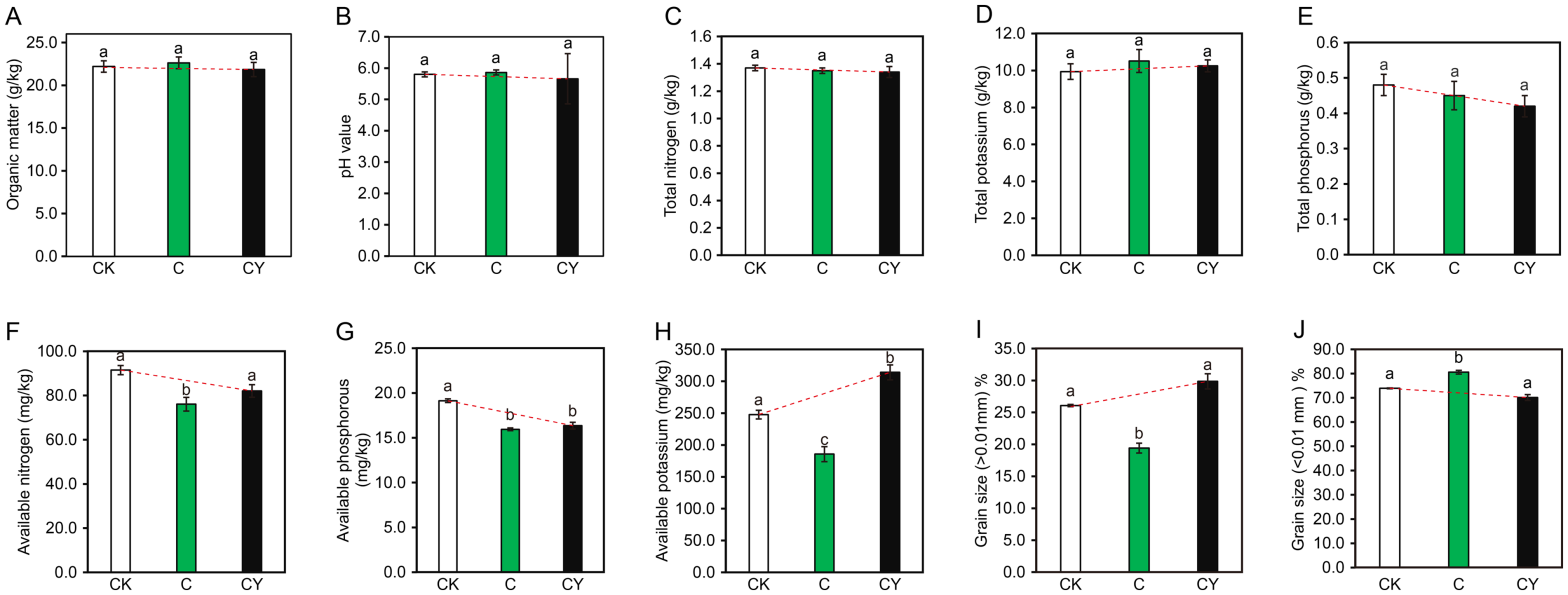
Impacts of various treatments on soil physicochemical properties. **(A)** Organic matter, **(B)** pH value, **(C)** total nitrogen, **(D)** total potassium, **(E)** total phosphorus, **(F)** available nitrogen, **(G)** available phosphorus, **(H)** available potassium, **(I)** grain size greater than 0.01 mm percentage, **(J)** grain size less than 0.01 mm percentage. Different lowercase letters after the same column data indicate significant differences among treatments (*p* < 0.05).

The physicochemical analysis of the soil (Figure 2) showed C_10_H_5_Cl_2_NO_2_ stress significantly reduced available nitrogen, phosphorus, and potassium (C vs. CK; Figures 2F–H). Importantly, K_2_S_2_O₈ application (CY) counteracted these reductions, restoring available nitrogen and potassium to near-CK levels (Figures 2F,H). Soil particle analysis (Figures 2I–J) further revealed that C₁₀H₅Cl₂NO₂ altered granular structure (>0.01 mm vs. <0.01 mm), while K₂S₂O₈ rehabilitated proportions to CK-equivalent states. These results demonstrate K₂S₂O₈‘s dual capacity to alleviate herbicide damage and restore soil functionality.

### 3.3 Influence of K_2_S_2_O₈ and C_10_H_5_Cl_2_NO_2_ on rhizosphere microbial communities

Metagenomic sequencing of root-associated communities yielded 364,770,378 clean reads from nine samples (treatments C, CY, and the control CK; Supplementary Table 2). Assembly generated 1,133,605 contigs (N50 > 680 bp), with open reading frame (ORF) prediction identifying 2,265,851 ORFs, confirming dataset robustness for further analysis.

**Figure 3:**
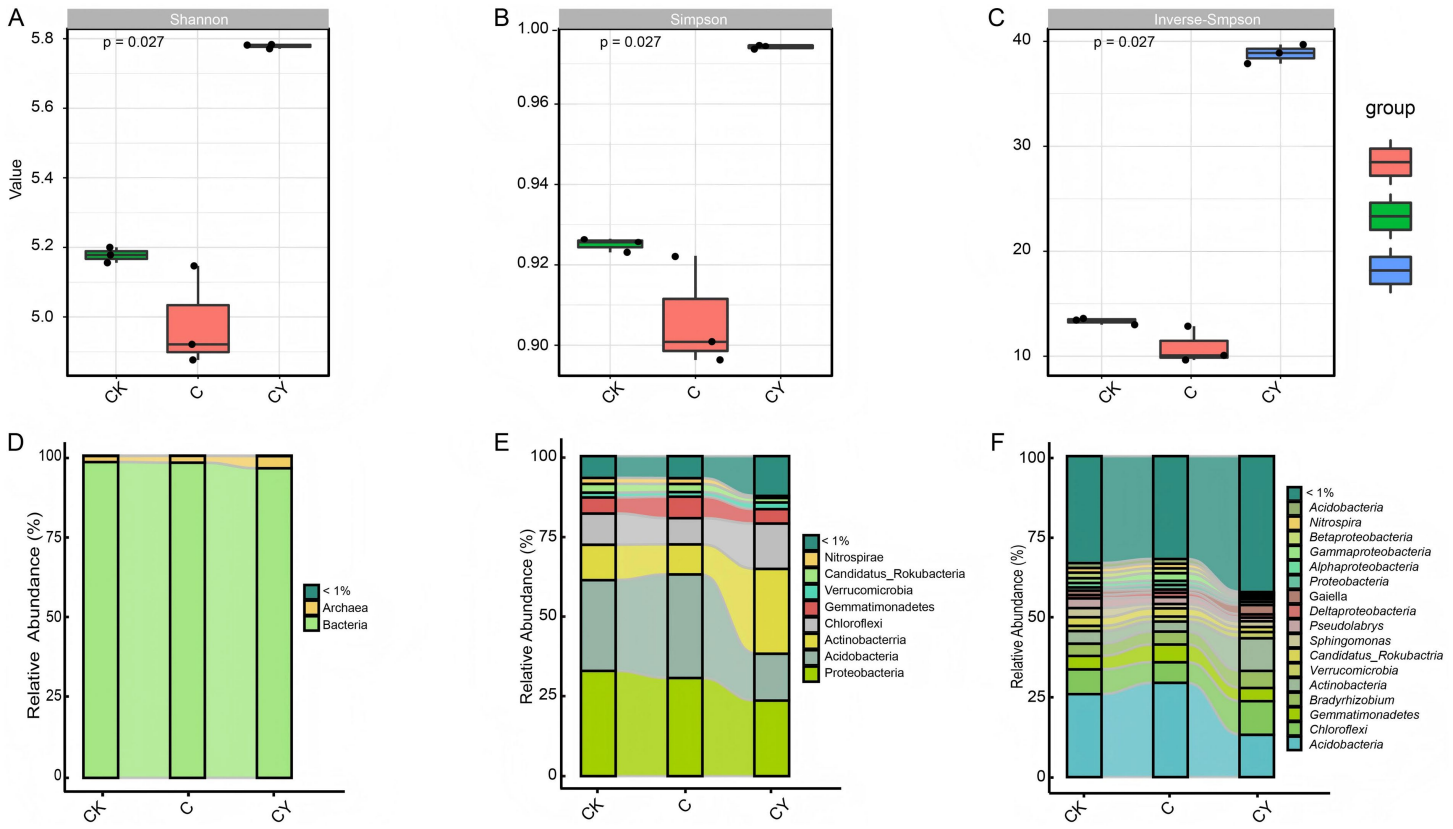
Investigation of rhizosphere microbial diversity. **(A)** Shannon index, **(B)** Simpson index **(C)** Inverse-Simpson index, **(D)** relative abundance at kingdom level, **(E)** relative abundance at phylum level, **(F)** relative abundance at genus level. The different substance categories are represented by different colors in the graph, showing substances that account for more than 1% of the total within each sample group, while the rest are categorized as <1%. The curves illustrate variations in reactant content among different samples.

Alpha diversity analysis using Shannon, Simpson, and Inverse-Simpson indices demonstrated that C_10_H_5_Cl_2_NO_2_ (C) exposure significantly reduced microbial diversity relative to CK. In contrast, K_2_S_2_O₈ (CY) amendment not only reversed this decline but enhanced diversity beyond control levels (Figures 3A–C), indicating effective mitigation of herbicide impacts on soil microbiota.

Taxonomic profiling (Supplementary Table 3) identified 4 kingdoms, 185 phyla, 305 classes, 495 orders, 928 families, 2,818 genera, and 12,641 species. Bacteria dominated microbial communities (96.05–97.99% relative abundance), with archaea constituting the remainder (Figure 3D). Five core bacterial phyla-Proteobacteria, Acidobacteria, Actinobacteria, Chloroflexi, and Gemmatimonadetes-collectively represented 83.42–87.29% of relative abundance across treatments (Figure 3E). Notably, C_10_H_5_Cl_2_NO_2_ reduced the abundance of beneficial genera including *Acidobacteria*, *Chloroflexi*, *Gemmatimonadetes*, *Bradyrhizobium*, *Actinobacteria*, *Verrucomicrobia*, *Candidatus-Rokubacteria*, and *Sphingomonas*, while K_2_S_2_O_8_ treatment uniquely restored their prevalence (Figure 3F). These genus-specific shifts substantiate K_2_S_2_O_8_’s capacity to rehabilitate functional soil microbiomes compromised by quinclorac stress.

### 3.4 Influence of K_2_S_2_O₈ and C_10_H_5_Cl_2_NO_2_ on gene expression profile and metabolites in tobacco leaves

**Figure 4:**
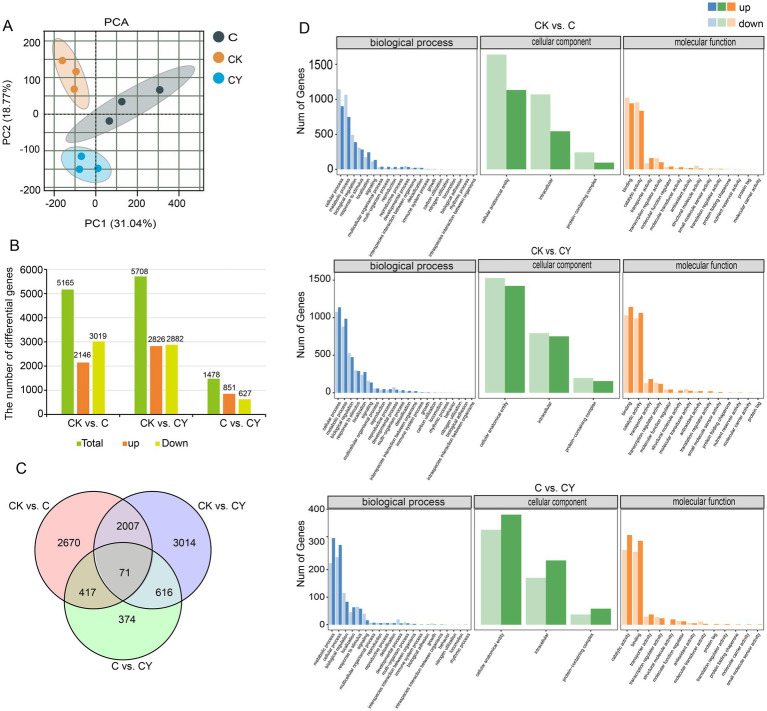
Transcriptomic analysis of samples. **(A)** PCA analysis of three treatment groups, **(B)** bar graph, and **(C)** Venn diagrams showing specific and common DEGs. The non-overlapping area of the Venn diagram represents the DEGs specific to the subgroup comparison, and the overlapping area represents the DEGs common to the several subgroup comparisons, **(D)** functional annotation of DEGs based on GO categorization.

To elucidate the mechanism by which K_2_S_2_O_8_ alleviates C_10_H_5_Cl_2_NO_2_ stress, we integrated transcriptomic and metabolomic analyses of tobacco leaves under CK, C, and CY treatments. PCA distinguished the C group from CK and CY (Figure 4A). Notably, C_10_H_5_Cl_2_NO_2_ stress (C vs. CK) induced 3,019 down-regulated and 2,146 up-regulated genes, while K_2_S_2_O_8_ supplementation (CY vs., C) reversed this trend, up-regulating 851 genes and down-regulating 627 genes (Figure 4B). Crucially, 71 DEGs were common across all comparisons (CK vs. C, CK vs. CY, C vs. CY; Figure 4C). Go enrichment confirmed that DEGs were primarily associated with stress response pathways, including cellular process (GO:0009987), metabolic process (GO:0008152), biological regulation (GO:0065007), localization (GO:0051179), response to stimulus (GO:0050896), signaling (GO:0023052). Critically, C_10_H_5_Cl_2_NO_2_ suppressed expression in these pathways (down-regulated > up-regulated in CK vs. C), while K_2_S_2_O_8_ restored expression levels, directly supporting its role in mitigating phytotoxicity (Figure 4D).

**Figure 5:**
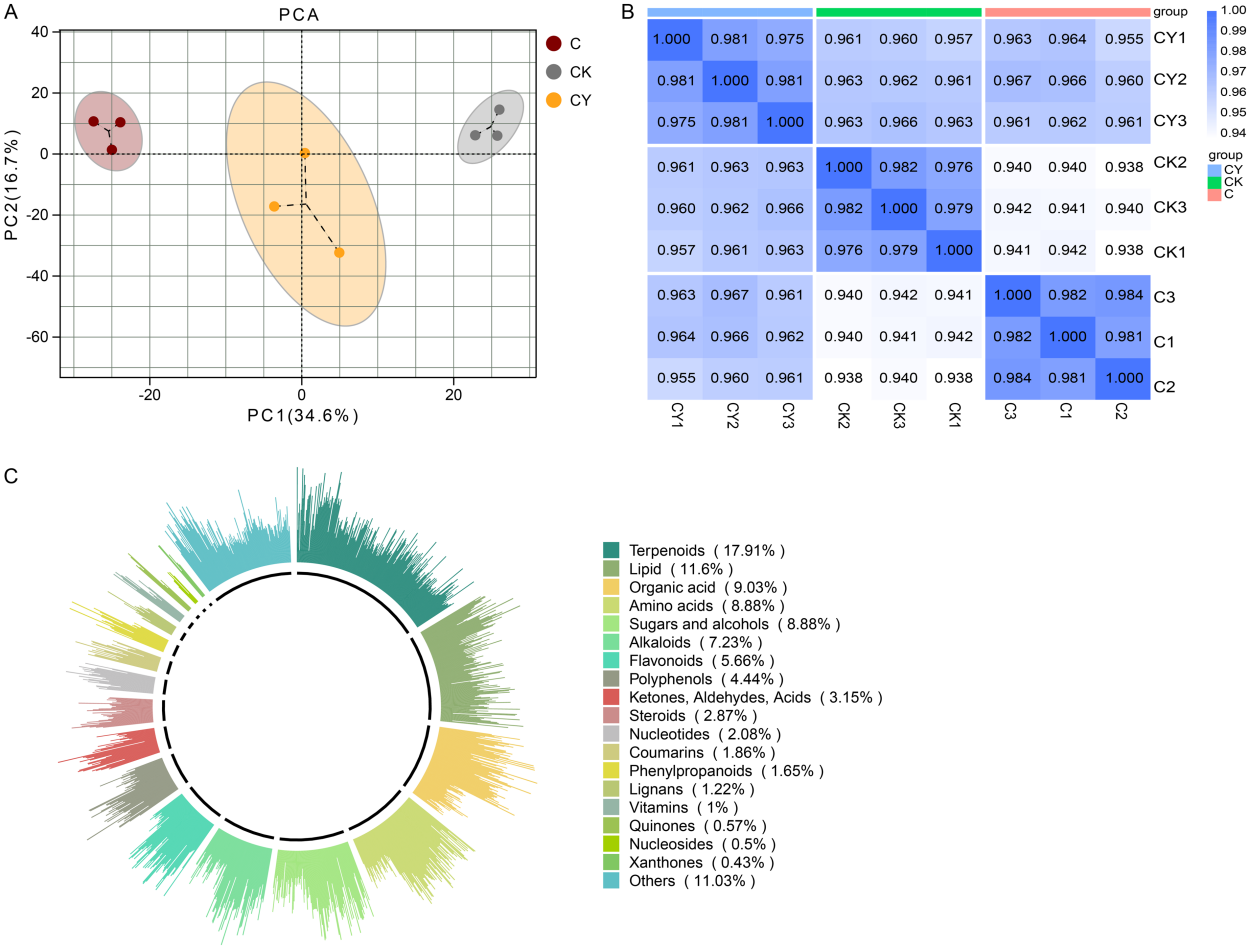
Analysis of the metabolite profiles of tobacco in three treatment groups. **(A)** Score scatter plot for principal component analysis (PCA) model. **(B)** Analysis of inter-sample correlations. **(C)** Classification of metabolites in the three treatment groups. The outermost circle of the figure illustrates various types of substances and their relative content. Each class of substances is represented by a specific color, while the length of each column indicates the proportionate content. In the second circle, the length of each line segment represents the proportion of classified substances within the total number. The longer the line segment, the greater number of substances falling under that classification.

Metabolite profiling revealed distinct clustering among CK, C, and CY groups (Figures 5A–C), validating data robustness. We identified 1,396 metabolites, dominated by terpenoids (17.9%), lipids (11.6%), organic acids (9.03%), sugars/alcohols (8.88%), and amino acids (8.88%) (Figure 5C).

**Figure 6:**
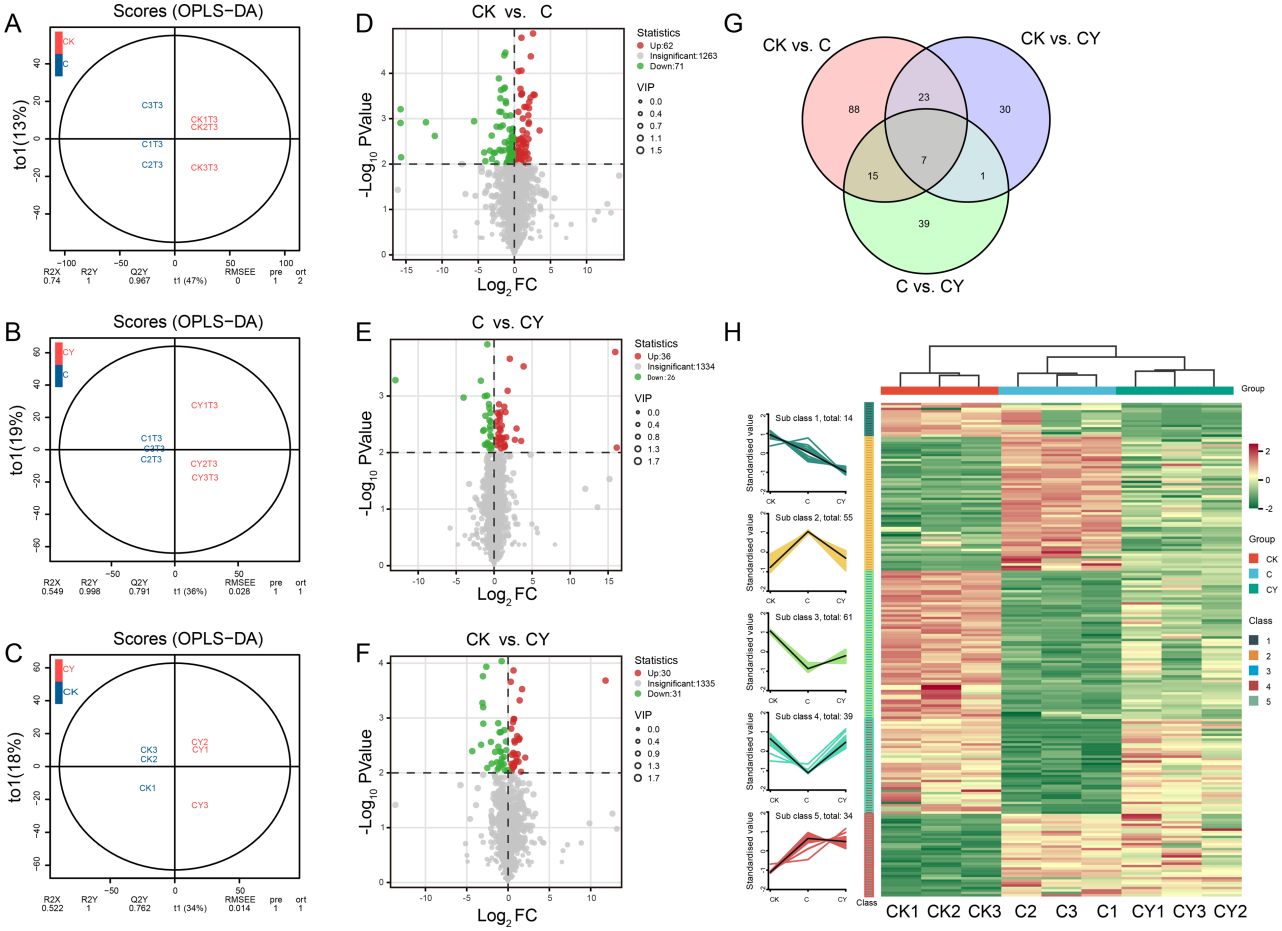
Differential expression metabolites analysis of samples. **(A–C)** OPLS-DA and **(D–F)** volcano plot analysis were performed for the comparisons of CK vs. C, C vs. CY, and CK vs. CY. **(G)** Venn diagram showing the numbers of common and specific DEMs among different comparisons. **(H)** Line graph and clustered heat-map visualization of significant differentially metabolites based on k-means clustering.

OPLS-DA confirmed significant inter-group differences (Figures 6A–C), and volcano plots quantified metabolites change: CK vs. C had 62 increased and 71 decreased metabolites, while C vs. CY showed 36 increased and 26 decreased metabolites, indicating K_2_S_2_O_8_’s normalization effect (Figures 6D–F). Among 203 differentially expressed metabolites (DEMs), seven were shared across all comparisons (Figure 6G). K-means clustering demonstrated that C_10_H_5_Cl_2_NO_2_ stress specifically depleted metabolites in cluster 3 and cluster 4 but elevated those in cluster 2 and cluster 5. Remarkably, K_2_S_2_O_8_ supplementation (CY) restored these metabolites to near-control (CK) levels (Figure 6H), highlighting its efficacy in rescuing stress-disrupted metabolic pathways.

### 3.5 Integrative analysis of metagenome, transcriptome, and metabolome

**Figure 7:**
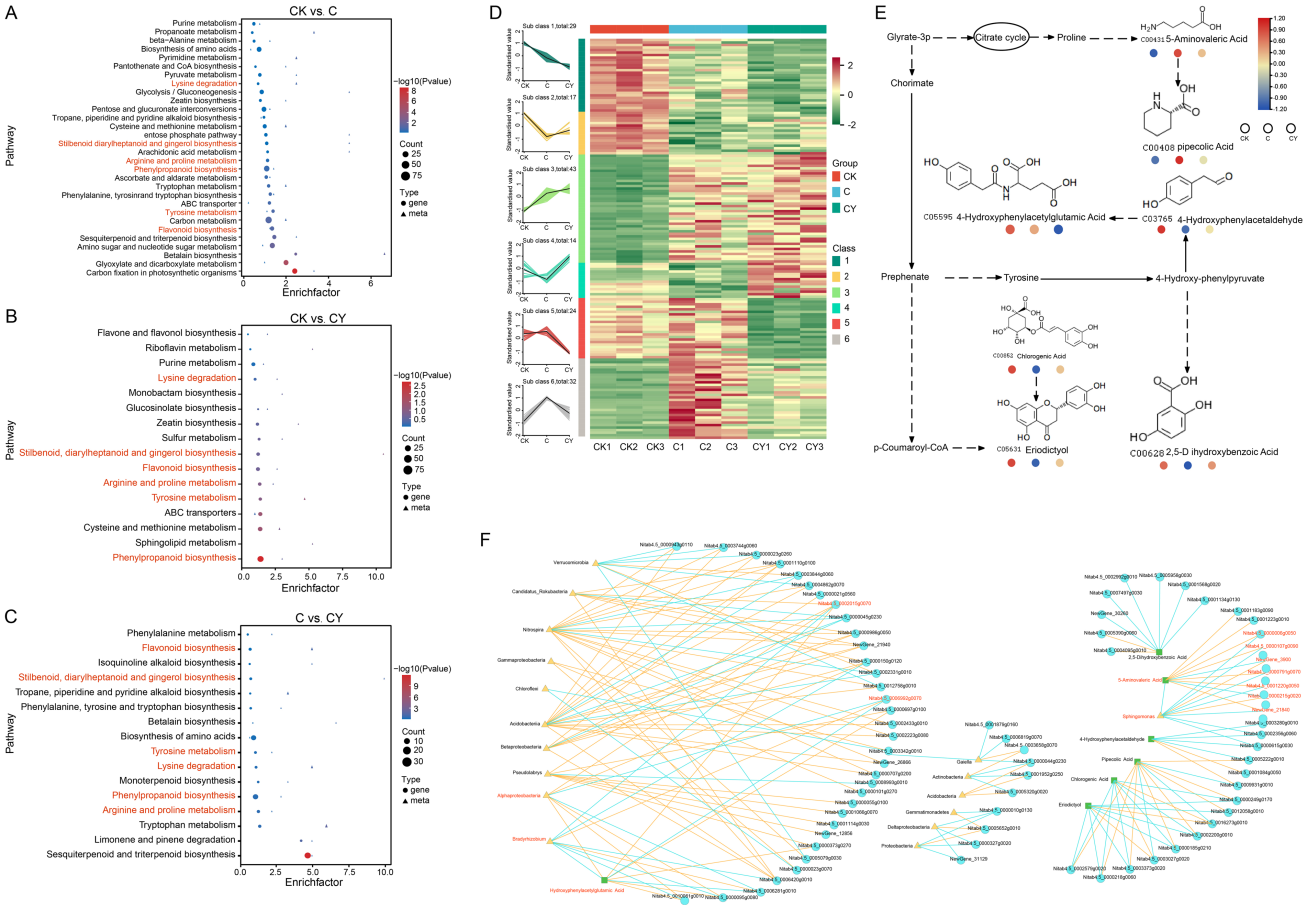
Association analysis of metabolome, transcriptome, and microbiome. **(A–C)** KEGG enrichment analysis for the DEGs and DEMs in three comparisons: CK vs. C, CK vs. CY, and C vs. CY. The x-axis represents the enrichment factor (Diff/Background) of different omics in this pathway, while the y-axis represents the names of KEGG pathways. The red-blue gradient indicates the degree of enrichment from high to low, as represented by *p*-value. The shape of bubbles represents different omics, and the size of bubbles represents the number of differential metabolites or genes, with larger bubbles indicating a greater quantity. **(D)** Line graph and clustered heat-map visualization of 159 significant differentially expressed genes based on k-means clustering. **(E)** Six shared metabolic pathways in the three comparison groups. **(F)** The correlation among 7 DEMs, 159 DEGs and the relative abundance (>1%) of microbial taxa at the genus level in three comparison groups. Triangles represent surface microbial genera, circles represent genes, square represent metabolites, and larger triangles, circles or squares indicate higher connectivity among genera, genes, and metabolites. Yellow lines represent positive correlations, while blue lines represent negative correlations.

To reveal the core metabolic pathways modulated by K₂S₂O₈ in alleviating C₁₀H₅Cl₂NO₂ stress, we conducted an integrative analysis of metagenome and transcriptome. KEGG analysis identified DEGs and DEMs co-enriched in six key metabolic pathways, including lysine degradation, stilbenoid diarylheptanoid and gingerol biosynthesis, arginine and proline metabolism, phenylalanine biosynthesis, tyrosine metabolism, and flavonoid biosynthesis (Figures 7A–C). A total of 159 DEGs were identified in the six common metabolic pathways, which were classified into 6 clusters by K-means analysis based on their similar expression patterns. Critically, C_10_H_5_Cl_2_NO_2_ stress (C vs. CK) significantly suppressed gene expression in cluster 2 and cluster 4, while inducing expression in cluster 6. Strikingly, K_2_S_2_O_8_ supplementation (CY) effectively reversed these stress-induced alterations, restoring expression levels in these clusters close to those observed in the control (CK) (Figure 7D). This restoration pattern strongly supports K₂S₂O₈’s role in counteracting C₁₀H₅Cl₂NO₂-induced dysregulation within these critical pathways. Additionally, compared to control (CK), genes in cluster 1 showed decreasing expression in both C and CY treatments, while genes in cluster 3 exhibited increasing expression in both treatments, though CY induced more pronounced changes than C alone. Among the seven DEMs identified in these six common metabolic pathways, most displayed significantly altered levels under C₁₀H₅Cl₂NO₂ stress (CK vs. C) but were restored towards control levels by K₂S₂O₈ supplementation (CK vs. C, CK vs. CY) (Figure 7E).

A multi-omics correlation network highlights K₂S₂O₈-mediated regulatory interactions. To elucidate the interplay among metabolomics, transcriptomics, and microbiota, we constructed a correlation network comprising 7 common DEMs, 159 DEGs, and microbial taxa with relative abundance greater than 1% (Figure 7F). The network consists of 79 nodes and 173 edges (96 positive, 77 negative). This included gene-microbe (50 nodes, 112 edges), gene-metabolite (18 nodes, 22 edges), and microbe-metabolite (2 nodes, 3 edges) interactions. Notably, a significant negative correlation was identified between the metabolite 5-Aminovaleric Acid and the microbe *Sphingomonas* regulated by seven genes (*NewGene_3900*, *Nitab4.5_0000215g0020*, *Nitab4.5_0000791g0070*, *Nitab4.5_0000006g0050*, *Nitab4.5_0001220g0050*, *Nitab4.5_0000107g0090*, and *NewGene_21840*). Furthermore, 4-Hydroxyphenyacetylgultamic Acid exhibited a significant negative correlation with *Bradyrhizobium* and *Alphaproteobacteria* regulated by *Nitab_4.50002015 g00700* and *Nitab450006992g00700*, respectively. These specific regulatory axes underscore the complex interplay between the microbiome, gene expression, and metabolite levels potentially modulated by K₂S₂O₈ in mitigating stress.

### 3.6 qRT-PCR validation

**Figure 8:**
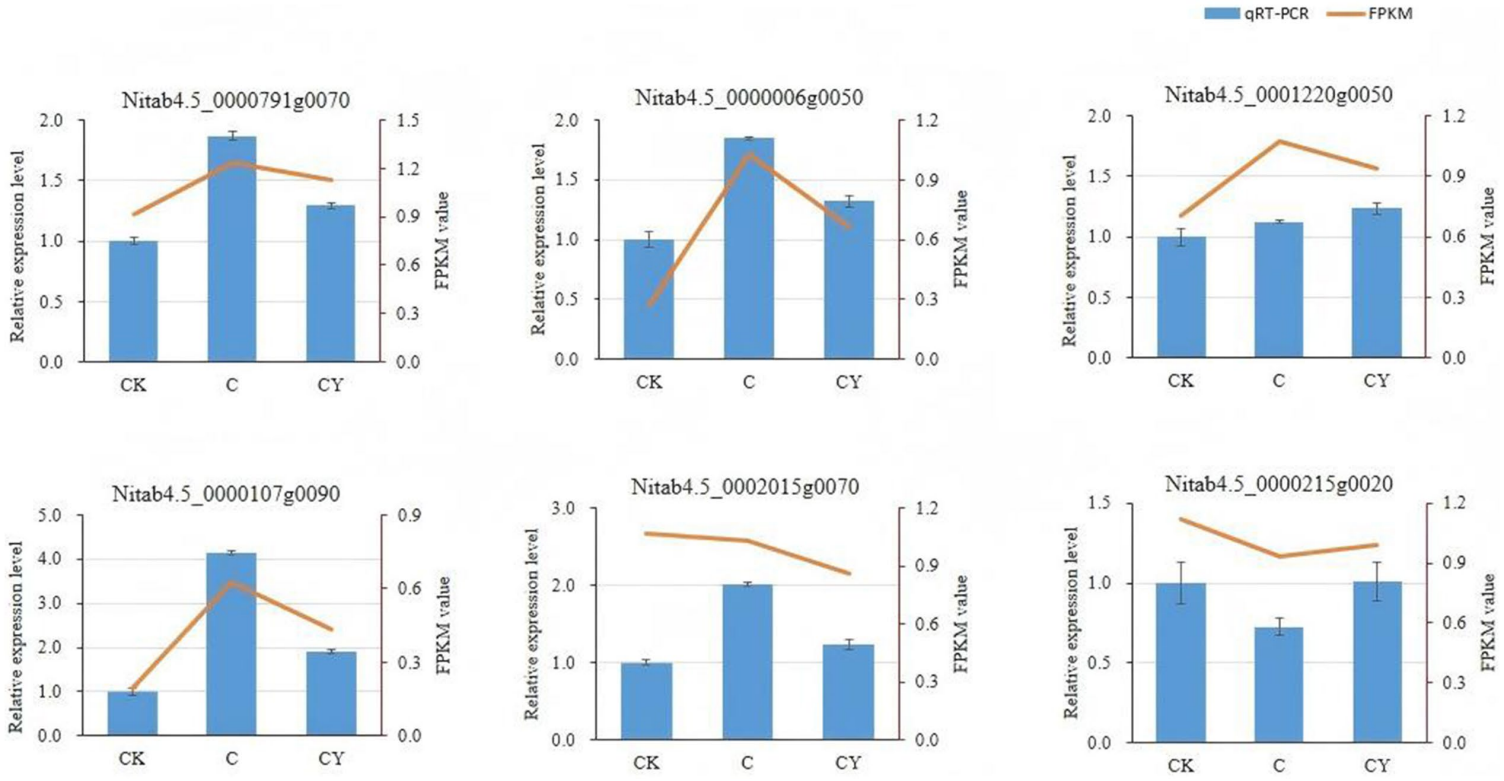
Relative expression level and FPKM of 6 genes in response to different treatment. Error bars represent standard deviations of three biological replicates, FPKM values were transformed by row scaling and log10 (*n* + 1), where *n* = FPKM values.

To verify the reliability of the transcriptome data, we selected six genes that showed significant correlations with both the metabolome and microbiome for qPCR validation. Compared with the CK group, the C and CY groups showed consistent trends, with four genes up-regulated and two genes down-regulated. Notably, the gene expression profile of the CY group was more closely aligned with that of the CK group compared to the C group. This observation serves as further evidence of the efficacy of the K_2_S_2_O_8_ treatment. The expression patterns of these six genes obtained through qRT-PCR were highly consistent with those from RNA-seq (Figure 8), confirming the reliability of the transcriptome-based differential gene expression analysis.

## 4 Discussion

Chemical oxidation remediation technology involves the utilization of chemical oxidants to expedite the degradation of pollutants in soil. This technology presents more advantages compared to physical remediation and bioremediation (Dermont et al., 2008), and it has demonstrated extensive application prospects in the field of contaminated site remediation. When compared with conventional oxidants such as Fenton reagent, ozone, and KMnO₄, persulfate-based strategies offer a superior redox potential and a longer half-life in soil matrices (Yen et al., 2011; Zeng et al., 2016). Previous studies have primarily focused on the degradation of polycyclic aromatic hydrocarbons (PAHs) (Chen et al., 2016) and atrazine (Chen et al., 2018) by persulfate. In this study, we innovatively integrated physiological, biochemical, and multi-omics analysis methods. We comprehensively investigated the effects of potassium persulfate (K₂S₂O₈) on remediating quinclorac-contaminated soil from multiple perspectives, including the impact of the oxidant on plant phenotypes, soil physicochemical properties, and the environmental micro-ecosystem. The results showed that oxidant K₂S₂O₈ can successfully remediate quinclorac-contaminated soil. It not only mitigates quinclorac-induced phytotoxicity, but also replenishes essential soil nutrients (nitrogen, phosphorus, potassium) that are depleted under quinclorac stress (Figure 2). This recovery of soil fertility is vital for sustainable agricultural practices (Daniel and Bernot, 2014), thereby providing new perspectives on addressing a critical challenge in the soil remediation paradigms for rice-tobacco rotation systems.

Secondary metabolic pathways play a crucial role in enabling plants to survive non-biological stress by regulating the levels of secondary metabolites and related gene expression (Lasky et al., 2014). This study integrated transcriptomic and metabolomic analyses to reveal that the DEGs and DEMs identified under quinclorac stress and K₂S₂O₈-mediated stress mitigation were enriched in six metabolic pathways: diphenyl ethylene diterpenoid biosynthesis, gingerol biosynthesis, arginine and proline metabolism, phenylalanine biosynthesis, tyrosine metabolism, and flavonoid biosynthesis (Figures 7A–C). Of these metabolic pathways, the arginine and proline metabolism directly link with ethylene synthesis through their competition for the common precursor S-adenosylmethionine (SAM), which is essential for both pathways, and through the regulatory interactions that influence the expression of key genes involved in each process (Zhao et al., 2024). All these pathways play a crucial role for regulating secondary metabolites and other protective mechanisms (Arruda and Barreto, 2020; Batista-Silva et al., 2019; Chong et al., 2009; Landi and Gould, 2015; Sharma et al., 2019; Tzin and Galili, 2010). Furthermore, most of the DEGs and DEMs (Figures 7D,E) involved in these pathways exhibited more similar expression levels in the comparison of CK vs. CY treatment compared to CK vs. C, indicating that K₂S₂O₈ application reversed the suppression of these pathways, largely restored the expression of most genes and the levels of key metabolites in these pathways towards those observed in the control. Moreover, it is reported that quinclorac can act as an auxin agonist to activate auxin signaling pathways in plants, leading to growth regulation and inhibition in susceptible species such as tobacco (Song et al., 2022). In this study, we found that at least eight auxin response factor or related genes were up-regulated in CK vs. C group, but down-regulated in C vs. CY treatment, which indicates that K₂S₂O₈ treatment reduces the interference of quinclorac on hormone signal transduction.

Quinclorac exposure significantly reduced microbial diversity, disrupting the balance of beneficial taxa such as Acidobacteria, Gemmatimonadetes, Actinobacteria and Chloroflexi. However, K₂S₂O₈ addition remarkably enhanced microbial richness and restored key beneficial genera such as *Sphingomonas* and *Bradyrhizobium* (Figure 3). Multi-omics network analysis elucidated the theoretical implications of these microbial shifts, revealing strong correlations between the restored genera (*Sphingomonas*, *Bradyrhizobium*), key metabolites (5-aminovaleric acid, 4-hydroxyphenylacetylglutamic acid), and differentially expressed genes (DEGs; e.g., *Nitab4.5_0000215g0020*, *Nitab4.5_0000791g0070*) (Figure 7F). These metabolites played a central role in the intricate interplay among soil properties, microbial communities, and plant health—fundamental to agricultural sustainability (Rybnikova et al., 2017). This demonstrates how K₂S₂O₈ reestablishes critical ecological interactions by mitigating quinclorac-induced disruptions to soil structure, nutrient availability, and microbial diversity. The results indicate that K₂S₂O₈ fosters a beneficial microbial environment, which crucially modulates plant stress responses through lysine degradation and flavonoid biosynthesis pathways—a novel mechanistic synergy in herbicide remediation. Based on these findings, a hypothetical model was proposed to illustrate the mechanism by which K₂S₂O₈ alleviates herbicide damage (Supplementary Figure S1). This enhancement potentially improves nutrient cycling and mitigates phytotoxicity (Sessitsch and Mitter, 2015; Wu et al., 2021), while emphasizing the utility of multi-omics techniques in exploring such complex ecological relationships (Qian et al., 2015; Timmusk and Wagner, 1999). However, more specific mechanistic insights require further in-depth research to fully elucidate the underlying processes.

Quinclorac is an auxinic herbicide widely used to control monocotyledonous weeds, particularly in rice cultivation systems. However, its persistence in acidic soils has raised significant concerns due to its phytotoxicity toward subsequent crops, especially *Solanaceous* species such as tobacco, potato, tomato, and eggplant (Grossmann, 1998; Qiuzan et al., 2018). It is reported to be absorbed by plant roots and transported to shoots, where it induces ethylene and cyanide production, alters plant hormone levels, and causes oxidative stress, ultimately inhibiting growth in sensitive plants (Song et al., 2022). In this study, we demonstrated that quinclorac exposure led to severe growth inhibition in tobacco seedlings, evident through deformities in leaves and roots. This finding is consistent with previous reports that have highlighted the adverse effects of the herbicide on crop health and yield (Huang et al., 2021). When K₂S₂O₈ was introduced into the soil contaminated with quinclorac, it effectively mitigated these detrimental impacts. The crops in the treated soil were able to resume normal growth patterns, which strongly underscores the potential of K₂S₂O₈ as a promising remediation agent for quinclorac-contaminated soil. This outcome vividly demonstrates the practical feasibility of the method. Notably, when applying this method in field environments, a comprehensive consideration of numerous complex factors is necessary. These factors encompass the soil’s pH value, its physical and chemical properties, the soil microecological environment, diverse climatic conditions, and the cost of implementation. To reduce costs, minimize environmental impact, and effectively reduce phytotoxicity of the herbicide, we employed a “hole application” method in our field experiments. Before tobacco transplantation, selectively treated only the planting holes and the adjacent soil of the plants with K₂S₂O₈. This targeted approach not only minimizes resource utilization but also reduces potential negative impacts on the broader environment. The results clearly demonstrated that following the K₂S₂O₈ treatment, the plants showed superior growth, and their root systems were more robustly developed throughout both the seedling and mature stages (Supplementary Figure S2). Undoubtedly, additional in-depth exploration is required to formulate a more comprehensive and optimized utilization method for this treatment. Future field-scale studies should carefully address spatial heterogeneity, the effects of rainfall, and long-term microbiome resilience to further enhance soil remediation strategies. This will allow us to fully realize its potential and maximize its benefits in agricultural practices.

## 5 Conclusion

This study demonstrates that the oxidizing agent K₂S₂O₈ effectively mitigates the adverse effects of quinclorac herbicide on both agricultural soil and tobacco plants. The integration of biochemical, metagenomic, metabolomic, and transcriptomic analyses demonstrated that K₂S₂O₈ significantly mitigated the quinclorac-induced alterations in gene expression and metabolite profiles, bringing them close to control level. Specifically, K₂S₂O₈ increases the abundance of beneficial microbial flora such as *Sphingomonas* and *Bradyrhizobium*, while decreasing harmful bacteria. Additionally, it modulates key metabolic pathways affected by quinclorac, such as arginine and proline metabolism, lysine degradation, and flavonoid biosynthesis. Furthermore, K₂S₂O₈ suppresses the quinclorac-induced increase in auxin response factor and related genes, thereby mitigating its interference with hormone signal transduction. This research offers a comprehensive approach to remediate pesticide-contaminated soils in rice–tobacco rotation systems, supporting sustainable agricultural practices.

## Data Availability

The datasets presented in this study can be found in online repositories. The names of the repository/repositories and accession number(s) can be found in the article/Supplementary material.

## Footnotes

^1^ www.biocloud.net

^2^ https://solgenomics.net/organism/Nicotiana_attenuata/genome

^3^ https://www.kegg.jp/kegg/compound/

^4^ https://www.kegg.jp/kegg/pathway.html
