# Supplementary figures and images for: Multi-omics analysis reveals the alleviating effect of oxidation remediation on tobacco quinclorac stress
Source: Front Microbiol. 2025 Sep 15;16:1625585. doi: 10.3389/fmicb.2025.1625585 (PMC12477241; doi:10.3389/fmicb.2025.1625585)

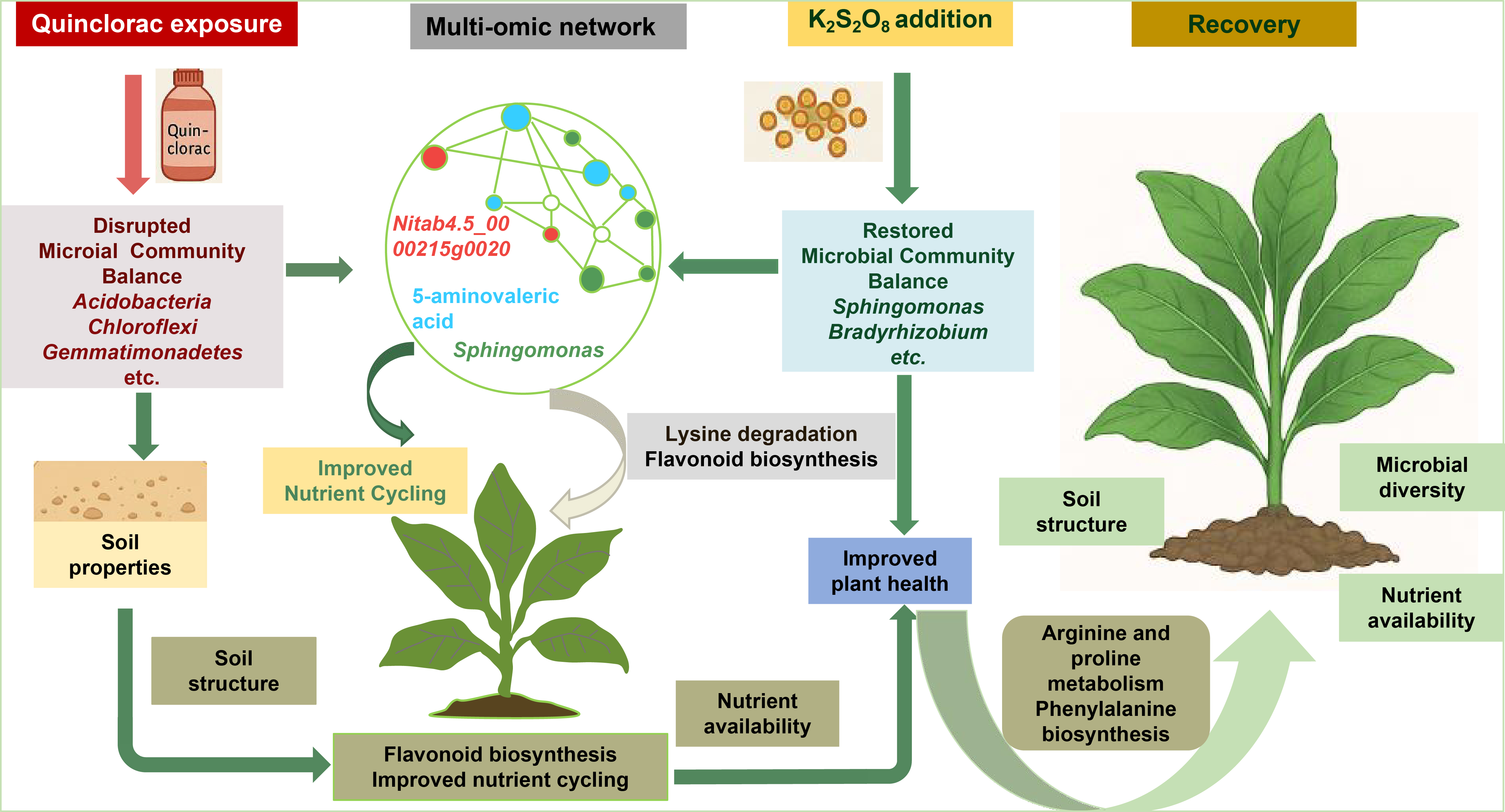

Supplement: Supplementary file 1 [file Image_1.JPEG]

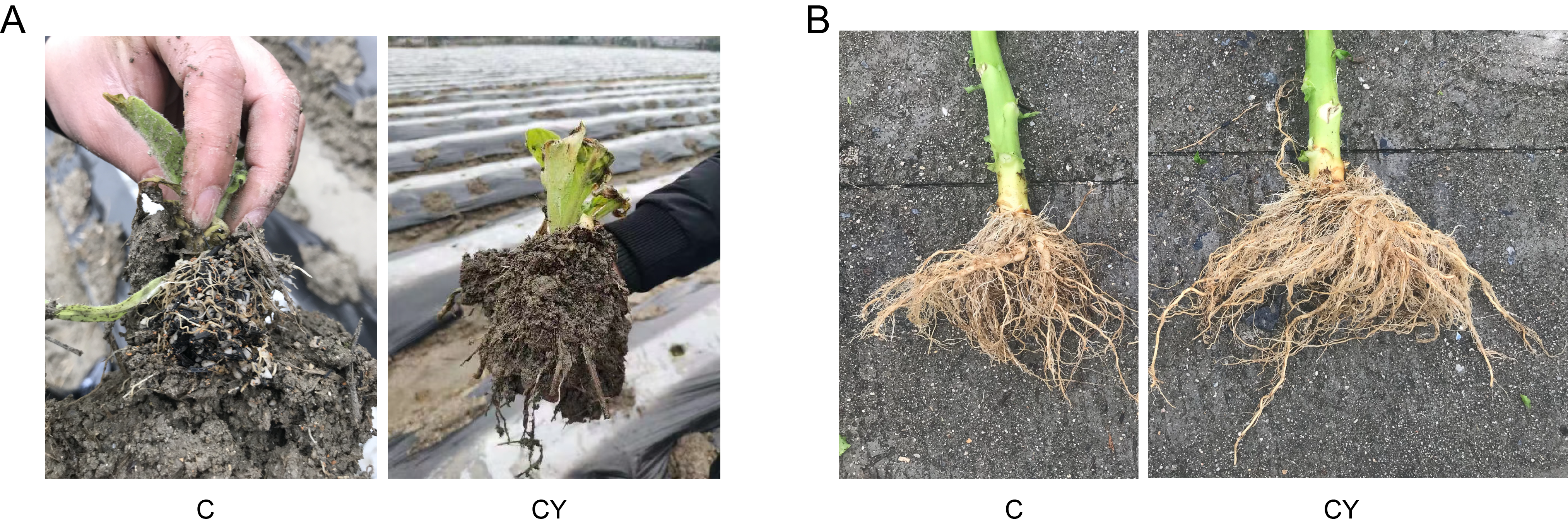

Supplement: Supplementary file 2 [file Image_2.JPEG]
